# Trophic Antioxidant Transfer as a Measure for Scalable Coral Conservation Strategies

**DOI:** 10.3390/antiox15080994

**Published:** 2026-08-11

**Authors:** Juan José Dorantes-Aranda, Cécile Rottier, Emma F. Camp, Jennifer L. Matthews, Christine Ferrier-Pagès

**Affiliations:** 1 Coral Biology Team, Centre Scientifique de Monaco, 98000 Monaco, Monacoferrier@centrescientifique.mc (C.F.-P.); 2 Climate Change Cluster, University of Technology Sydney, Sydney, NSW 2007, Australia; emma.camp@uts.edu.au (E.F.C.); jennifer.matthews@uts.edu.au (J.L.M.)

**Keywords:** coral bleaching, conservation, antioxidant defence, micronutrients, thermal stress, oxidative stress, curcumin, *Pavlova lutheri*

## Abstract

Coral reefs are increasingly threatened by elevated seawater temperatures associated with heatwaves and El Niño events. These phenomena challenge conventional management practices, highlighting an urgent need for innovative interventions to enhance reef resilience. Investigating the role of oxidative stress—one of the main explanations for coral bleaching—is central to these efforts, as excessive production of reactive oxygen species can impair coral physiology, disrupt the coral–algal symbiosis, and ultimately lead to mortality. Here, we investigated an antioxidant-rich food web approach by feeding the reef-building coral *Stylophora pistillata* with *Artemia* that had been pre-fed with (i) low-cost and in-house-produced pellets containing curcumin, fucoxanthin, astaxanthin, vitamins C or E, or (ii) the phytoplankton species *Pavlova lutheri*, *Symbiodinium* sp., *Nannochloropsis* sp., *Dunaliella salina* or *Synechococcus* sp. Curcumin and the microalga *P. lutheri* offered the best protection to corals against oxidative stress, and represent the best candidates for potential scalable coral conservation interventions involving targeted feeding of antioxidants. Curcumin pellets offer a streamlined alternative to *P. lutheri* by eliminating the need for multi-stage microalgal culturing. Direct enrichment of *Artemia* with antioxidant-rich pellets simplifies production and may provide a cost-effective, scalable strategy to enhance coral resilience to oxidative stress, bleaching and potential mortality.

## 1. Introduction

Coral reefs face escalating threats from climate change, particularly due to the increasing occurrence of marine heatwaves that can induce severe thermal stress [1]. The global coral crisis is further intensified by local and regional stressors, including poor water quality, cyclones, overfishing, coral predators (e.g., crown of thorns starfish) and emerging diseases (e.g., stony coral tissue loss disease) [2,3]. Under thermal stress conditions, both the coral host and its symbiont microalgae (Symbiodiniaceae) tend to produce high levels of reactive oxygen species (ROS) [4,5,6]. Sustained production of ROS can induce oxidative stress in both the coral host and its symbiotic microalgae, damaging cellular components and destabilising the coral–algal symbiosis. The breakdown of this symbiosis results in the loss of symbionts and/or their photosynthetic pigments, leading to coral bleaching [5,7]. Coral bleaching can have severe consequences because Symbiodiniaceae provide corals with more than 80% of their energetic requirements and give corals their characteristic colouration [8,9]. Bleaching has traditionally been explained by the oxidative stress theory, where excessive ROS production drives the breakdown of the coral–algal symbiosis [8,10,11,12]. More recently, the carbon limitation theory has proposed that thermal stress disrupts the translocation of photosynthetically derived carbon from Symbiodiniaceae to the coral host, resulting in energy limitation and, ultimately, host starvation [13,14]. Heatwaves also limit zooplankton availability, and therefore corals cannot easily compensate for the loss of autotrophy with heterotrophy [15,16]. These prolonged conditions of nutritional deprivation impair coral health and enhance susceptibility to diseases, ultimately leading to large-scale mortality events [17].

Urgent mitigation of climate change impacts, such as achieving the goal of global net zero emissions in accordance with the Paris Agreement on Climate Change [18,19], is necessary to ensure the long-term future of coral reefs. However, to buy time for coral reefs while global efforts to reduce greenhouse gas emissions continue, a growing toolbox of reef interventions has been developed to enhance coral resilience and support ecosystem recovery. These interventions include, but are not limited to, coral propagation and transplantation, assisted evolution, selective breeding, symbiont manipulation, and nutritional supplementation. Collectively, these approaches seek to improve coral performance under environmental stress while maintaining genetic diversity and adaptive capacity [18,20,21]. While restoring degraded coral reefs is essential, protecting healthy reefs before significant decline occurs is equally important. Consequently, several active in situ interventions have been developed to increase coral resilience during periods of environmental stress. These include shading and cooling technologies designed to create local thermal refugia [22], the use of artificial lighting to enhance coral feeding and energy acquisition [23], and the application of beneficial microbial consortia (probiotics) cultured from healthy corals to improve host health and stress tolerance [24]. These probiotic bacteria possess infection resistance as well as ROS scavenging properties, thereby offering protection to corals against disease and oxidative stress to reduce bleaching and mortality [24,25,26]. In parallel, nutritional interventions have demonstrated considerable promise in coral aquaculture, where heterotrophic feeding enhances growth, energy reserves, reproduction, and recovery from environmental stress [27]. Targeted nutritional supplementation has also been shown to improve survival during vulnerable early life stages, highlighting nutrition as a viable pathway for enhancing coral resilience across multiple life-history stages [28]. The search for novel coral conservation interventions has identified manganese and curcumin as promising antioxidant supplements capable of mitigating oxidative stress and reducing bleaching under thermal stress [29,30,31,32]. While these approaches have shown potential in experimental settings, their practical application at reef scales remains challenging due to limitations in delivery, logistics, and cost. Consequently, alternative delivery pathways that are scalable and operationally feasible are needed. Leveraging natural feeding behaviour to administer targeted nutritional supplements offers one such approach, providing an efficient mechanism to deliver bioactive compounds while simultaneously supporting coral energetic demands. However, the potential of nutritional supplementation as a practical reef-scale intervention remains largely unexplored [33].

The use of antioxidant supplements is currently a common practice in aquaculture, just as in human health and terrestrial animal farming. The most popular antioxidants include carotenoids, vitamins C and E, flavonoids and polyphenols (reviewed by Hu et al. [34]). Their effects in aquatic organisms include protection against oxidative stress and improvement in immune function and reproductive and growth performance [34,35,36]. Moreover, some of these antioxidants are also pigments; therefore, their use in aquaculture provides a two-fold purpose since colouration in fish is favoured using carotenoids such as astaxanthin and polyphenols such as curcumin [35,37,38]. Several phytoplankton species produce antioxidant pigments *de novo*, making them good candidates for the commercial production of antioxidants, especially when triggering a higher antioxidant production by means of nutrient limitation, temperature or light stress [39]. However, scaling these approaches from the laboratory to reef applications would require the mass culture of antioxidant-producing microorganisms, a process that can involve considerable infrastructure, investment, and operational costs [40,41]. Therefore, aquaculture practices commonly employ a food web approach, utilising live feed such as *Artemia*. This and other zooplankton are reared and fed with nutrient-rich phytoplankton to accumulate essential nutrients that can be transferred to higher trophic levels, such as fish and crustaceans [39,42,43]. Inspired by these established aquaculture practices, we investigated whether antioxidant-enriched live feed could be used as a practical delivery platform for coral conservation, enabling the transfer of bioactive compounds to corals through their natural heterotrophic feeding behaviour.

In the present study, the coral *Stylophora pistillata* was exposed to heat stress (from 26 to 33 °C) and provided *Artemia salina* as a heterotrophic food source. The *Artemia* was first enriched with either live phytoplankton, or low-cost, in-house-formulated antioxidant-rich pellets. Following the three-week experimental period, photosynthetic efficiency, bleaching responses, and oxidative stress biomarkers were measured to assess the physiological effects of antioxidant supplementation on corals exposed to heat stress. We identified two strong candidates for targeted feeding-based coral conservation interventions, as both effectively alleviated oxidative stress and mitigated bleaching in corals under heat stress.

## 2. Material and Methods

### 2.1. Preparation of Coral Nubbins

One hundred and forty fragments of the scleractinian coral *Stylophora pistillata* (2–3 cm long) were obtained from three large mother colonies maintained at the Centre Scientifique de Monaco (CSM; originally collected from the Red Sea; CITES permits No. NL024463A011 & FR0808101265). These coral nubbins were acclimatised for three weeks in 20 L aquaria using an open system (seawater renewal at a flow rate of 8 L h^−1^). The seawater supplying the aquaria was pumped from a depth of 50 m and prefiltered using a sand filter and a sock-type filtration system (100 and 20 µm), followed by filtration through activated carbon, and finally passed through a UV lamp before entering the aquaria. The salinity of the seawater was 37 ± 1 g kg^−1^ and the water temperature was maintained at 26 ± 1 °C using submersible heaters (300 W Aqua Medic, Bissendorf, Germany). Light was supplied using a PAR LED module (Radiometrix LED GR2, Alpheus, Montgeron, France) at an intensity of 200 ± 15 µmol photons m^−2^ s^−1^ and with a photoperiod of 12 h:12 h dark:light. The water heaters and the light modules were connected to an automated controlling system (Enoleo Monaco, Monaco) to ensure a constant temperature and photoperiod. Coral nubbins were fed with non-enriched *Artemia salina* nauplii twice per week during the acclimatisation period.

### 2.2. Feeding of Artemia salina with Antioxidant-Rich Pellets and Phytoplankton

#### 2.2.1. Preparation of Antioxidant-Rich Pellets

Five commercially available antioxidants were sourced to formulate pellets using in-house-developed recipes. These included vitamins C and E, and the three antioxidant pigments fucoxanthin, astaxanthin and curcumin. Vitamin C is a hydrophilic substance, and the other four antioxidants have lipophilic properties; therefore, milli-Q water and soybean oil (Sigma, Saint-Quentin-Fallavier, France, S7381) were used as solvents accordingly. To ensure uniformity, all the pellets shared the same base ingredients, with only the antioxidant type varying; control pellets contained all the ingredients but no antioxidant. Briefly, 1.25 g of carrageenan (Sigma, C1013) and 1.25 g of wheat starch (Sigma, S5127) were mixed manually for 2 min (dry ingredients). The antioxidants were dissolved or dispersed individually according to Table 1, added to the dry ingredients, and then mixed manually for 5 min using a spatula. Once mixed, the paste was transferred and dispersed in a flat container and allowed to dry overnight at room temperature and protected from light. The containers containing the paste preparation were put in an incubator the following day (60 °C, 1 h) to ensure complete dryness and hardening of the preparation. The preparations were broken manually into smaller pieces and transferred into a coffee grinder and processed for 5 min to obtain small pellets. The drying temperature was selected to ensure pellet hardening; however, because several antioxidants are heat-sensitive, pellets were dried under light-protected conditions and stored at −20 °C immediately after processing. Future optimisation should quantify antioxidant retention after pellet manufacture.

#### 2.2.2. Preparation of Marine Phytoplankton Cultures

Four species of microalgae and one cyanobacterium were grown in f/2 medium [44] at a salinity of 34 g kg^−1^, incubated at 20 °C and at a light intensity of 200 ± 15 µmol photons m^−2^ s^−1^, with a photoperiod of 12 h:12 h dark:light. The cyanobacterium *Synechococcus* sp. was obtained from the Culture Collection of Algae & Protozoa SAMS Limited, UK (strain CCAP1479/22). The microalga *Pavlova lutheri* was obtained from the Roscoff Culture Collection, France (strain RCC1537); the microalgae *Nannochloropsis* sp., *Dunaliella salina* and *Symbiodinium* sp. (formerly Clade A) were available at the Centre Scientifique de Monaco. *Symbiodinium* sp. was originally isolated from a coral fragment of *S. pistillata* at CSM and continuously cultured *ex hospite*. All phytoplankton species were used at day 14 (exponential growth phase) to be fed to *Artemia salina*.

#### 2.2.3. Artemia Feeding

*Artemia salina* eggs were hatched after 24 h of continuous aeration under light conditions. *Artemia* nauplii (72 h post-hatched) were fed with the phytoplankton species or the antioxidant-rich pellets mentioned above. A set of 1 L beakers received 350 mL of *Artemia* nauplii in seawater (269,000 nauplii L^−1^), 150 mL of filtered seawater (0.45 µm) and 200 mL of each phytoplankton culture. The phytoplankton concentrations were 3.8 × 10^6^ *Synechococcus* sp. cells mL^−1^, 6.2 × 10^6^ *P. lutheri* cells mL^−1^, 4.4 × 10^5^ *Nannochloropsis* sp. cells mL^−1^, 4.2 × 10^5^ *D. salina* cells mL^−1^, and 4.1 × 10^5^ *Symbiodinium* sp. cells mL^−1^. Another beaker with *Artemia* nauplii was unfed and used for the thermally stressed control group of the phytoplankton experimental component. Another set of 1 L beakers also received 350 mL of seawater with *Artemia* nauplii as above, 150 mL of filtered seawater, and 200 mL of seawater containing 0.5 g of antioxidant-rich pellets, or pellets without antioxidants for the thermally stressed control group (pellet experimental component). Both experimental components, phytoplankton and pellets with antioxidants, had a negative control group each, consisting of coral nubbins maintained at 26 °C and fed with unfed *Artemia* nauplii. *Artemia* nauplii were allowed to feed on the phytoplankton species or enriched pellets for 5 h under light conditions, and with continuous agitation using a magnet and a stirring plate. Aeration was not used to avoid oxidation of the antioxidants contained in the pellets and potential lysis of fragile phytoplankton cells. After the 5 h feeding period, *Artemia* nauplii were filtered through a 100 µm filter mesh, rinsed with filtered seawater and resuspended in 200 mL of filtered seawater (1883 nauplii mL^−1^). Aliquots of 10 mL were transferred into 15 mL plastic tubes and frozen at −20 °C until further use to feed the corals.

### 2.3. Thermal Stress Experiments

Coral nubbins were randomly distributed into fourteen aquaria (five nubbins per 20 L aquarium), corresponding to five experimental conditions and two control groups, all in duplicate. Another set of fourteen aquaria was prepared in a similar manner. One set of aquaria was used to feed corals with pellet-fed *Artemia*, and the other set was used to feed corals with phytoplankton-fed *Artemia*. Each set had its own control groups, with one maintained at 26 °C throughout the entire experiment (i.e., negative control or non-thermally stressed) and fed with non-enriched *Artemia* nauplii. The corals in the other control group were thermally stressed and fed with non-enriched *Artemia* (phytoplankton experimental component) or fed with *Artemia* that had been fed with pellets without antioxidants (pellet component). All the experimental groups, except for the negative control, were subjected to a temperature increase from 26 to 33 °C (1.0 °C per day) to challenge the coral nubbins to a heatwave scenario, similar to those observed in the Red Sea where our mother colonies of *S. pistillata* originated from [45,46,47,48]. Once the 33 °C temperature was reached, it was maintained for three days, then reduced to 31 °C (1.0 °C per day) and held for a further six days.

Coral nubbins were fed three times per week during the 3-week experimental period. For this purpose, the coral nubbins were transferred into 2 L beakers, and 1.8 L of their respective aquarium seawater was added. The beakers with coral nubbins were set up in a water bath set to the corresponding experimental temperature. Each experimental group received 20 mL of enriched or non-enriched *Artemia* nauplii (1883 nauplii mL^−1^). *Artemia* nauplii were maintained in suspension by using a magnet and a stirring plate set up in the water bath. A similar set up was used to feed the negative control in their corresponding aquaria, which were always maintained at 26 °C.

#### Photosynthesis and Respiration Parameters in Corals

Photosynthetic efficiency parameters including the relative electron transport rate (rETR) and the maximum quantum yield efficiency of photosystem II (*F_v_*/*F_m_*) were determined in five coral nubbins per condition using a dual Pulse Amplitude Modulation fluorometer (Dual-PAM 100; Heinz Walz, Effeltrich, Germany) according to Hoogenboom et al. [49]. Briefly, coral nubbins were dark-adapted in a chamber containing seawater for 15 min with a heater installed in the chamber to maintain the same experimental temperatures as those in the aquaria. A light curve was produced by creating light flashes within the range of 11–3247 µmol photons m^−2^ s^−1^ using an optic sensor positioned in contact with the coral nubbin.

Photosynthesis and respiration rates were measured using the same five coral nubbins per experimental condition. Briefly, nubbins were individually placed in 50 mL chambers (Plexiglass) filled with 0.45 µm filtered seawater and maintained at the corresponding experimental temperature. The seawater in the chambers was continuously mixed using a magnet stirrer. Dissolved oxygen concentration (DO) was monitored through an optical sensor placed on top of the chamber in direct contact with the seawater and connected to a computer (software Oxy4v2-30fb, version 2). Coral nubbins were maintained in the chambers for 10 min, followed by photosynthesis determination using a PAR LED module (200 µmol photons m^−2^ s^−1^; Radiometrix LED GR2, Alpheus France). Subsequently, coral nubbins were maintained in the same chambers and dark-adapted for 20 min, followed by respiration determination. Calibration was performed using filtered seawater containing sodium sulphate (0% DO) and oxygen-saturated seawater (100% DO).

Once the experiments were completed, all coral nubbins were stored individually at −80 °C for further analyses. Nubbins used for photosynthesis and respiration tests were used to assess coral bleaching parameters, including chlorophylls *a* and *c2* and symbiont dinoflagellate concentrations. The rest of the nubbins (i.e., those not used for photosynthesis and previously undisturbed, *n* = 5) were flash frozen in liquid nitrogen and stored at −80 °C for oxidative stress biomarker analyses.

### 2.4. Coral Bleaching Parameters

The tissue from frozen coral nubbins was removed from the skeleton using an airbrush and filtered seawater at room temperature. Subsequently, the recovered tissue was homogenised using a tissue homogeniser (Ultra-Turrax T25, IKA-Labortechnik; Fisher Scientific, Illkirch, France). Two aliquots were distributed into tubes to determine chlorophyll *a *and *c2* concentrations and symbiont density. Aliquots were stored until analysis (4 °C for symbionts and −20 °C for chlorophyll determination).

One set of samples was thawed and centrifuged (4 °C, 5000 *g*, 10 min) and the supernatant discarded. The pellet was resuspended in 2.5 mL of >99% acetone and stored at 4 °C protected from light for a 24 h extraction period. The samples were centrifuged (4 °C, 5000× *g*, 15 min) and the supernatant recovered. Chlorophylls *a *and *c2* were determined spectrophotometrically in 96-well plates using a Xenius SAFAS plate reader (λ = 616 & 663 nm; Monaco, software SP2000V7, version 7).

Using the other set of samples, a subsample of 10 µL was used to determine symbiont cell density using a Luna-FX7 automated cell counter (Logos Biosystems; Dutscher, Bernolsheim, France). The chlorophyll *a* and *c2* concentrations and symbiont density were normalised to the size of the coral nubbins with the surface area of the coral nubbin skeleton determined using the wax-dipping method [50]. Briefly, once the tissue and symbionts were extracted, coral skeletons were dried in the oven (60 °C, 24 h) and weighed. The coral skeletons were then dipped in melted paraffin maintained at 65 °C (P3558 Sigma), after which they were submerged in icy water to harden the paraffin. Once dry, the coral skeletons were weighed again. Surface area was determined using the paraffin wax method by converting the increase in skeleton weight after waxing to surface area using a calibration curve derived from coral skeletons of known surface area.

### 2.5. Oxidative Stress Biomarkers

The coral nubbins that were undisturbed during the experimental period (i.e., not used for photosynthesis measurements; *n* = 5) were used to determine the oxidative stress by assessing several biomarkers as described below. Frozen coral nubbins were thawed, and a section of the tissue was obtained from the apex area (1 cm^2^); phosphate-buffered saline was added to the tissue and sonicated (20 pulses, 70%). The extract was centrifuged (4 °C, 11,000× *g*, 10 min) and the supernatant recovered and centrifuged again under the same conditions. Protein concentration was quantified in triplicate using the Bradford spectrophotometric method (λ = 595 nm) with bovine serum albumin used for the standard curve. All the extracts were harmonised to 1 mg protein mL^−1^ and used to determine the activity of the antioxidant enzymes catalase, glutathione peroxidase, and superoxide dismutase, as well as the total antioxidant capacity.

#### 2.5.1. Catalase

The activity of the enzyme catalase (CAT) was determined using a 96-well plate-based commercial kit (Invitrogen, EIACATC; Fisher Scientific, Illkirch, France). This assay relies on the consumption or hydrolysis of hydrogen peroxide by the catalase present in the sample, which is reflected by the change in absorbance. Samples were added to the wells in triplicate, followed by addition of hydrogen peroxide and incubation for 30 min at room temperature. Once the incubation period was completed, the supplied substrate solution was added to the wells, followed by the horseradish peroxidase solution and an extra 15 min incubation period at room temperature. The absorbance was measured at 560 nm, and the enzyme activity was calculated from a calibration curve using the catalase standard supplied by the manufacturer.

#### 2.5.2. Glutathione Peroxidase

The enzyme glutathione peroxidase (GPx) was assessed using a commercial kit (Sigma, MAK437) in a 96-well microplate format. This test measures the consumption of NADPH with the decrease in signal directly proportional to the activity of the enzyme. Samples were added to the wells in triplicate, mixed with the assay buffer and working reagent followed by addition of the substrate solution. The optical density (λ = 340 nm) was measured immediately after mixing the cocktail, followed by a 4 min incubation period and then measurement of the optical density again.

#### 2.5.3. Superoxide Dismutase

The activity of the enzyme superoxide dismutase (SOD) was quantified using a spectrophotometric 96-well plate-based assay that measures all types of SOD. The protocol provided by the manufacturer (Invitrogen, EIASODC; Fisher Scientific, Illkirch, France) was followed with sample extracts added to the microplate wells in triplicate and mixed with the provided SOD substrate and xanthine oxidase. The plate containing the cocktail was incubated for 20 min at room temperature. A standard curve was created using a bovine erythrocyte SOD standard (freshly prepared). The absorbance signal was measured at 450 nm.

#### 2.5.4. Total Antioxidant Capacity

The total antioxidant capacity (TAC) of the coral extracts was determined using a commercial kit (Cell Biolabs Inc., OxiSelect STA-360, Nanterre, France) with the assay based on the production of uric acid in the presence of copper. Briefly, extracts were added to the 96-well plate in duplicate, followed by addition of the reaction buffer and the copper solution (reaction starter). The microplate was incubated at room temperature for 5 min in an orbital shaker (400 rpm). Subsequently, the stop solution was added to all the wells and the absorbance measured at 490 nm. The standard curve was generated using uric acid.

#### 2.5.5. Lipid Peroxidation

The thiobarbituric acid reactive substance (TBARS) method was used to determine lipid peroxidation (LPO) in the coral extracts. This assay is based on the reaction of malondialdehyde (MDA, one of the byproducts and the main marker for LPO) with thiobarbituric acid (TBA) [51]. Briefly, coral fragments (1 cm^2^) were sonicated and homogenised in a solution containing 1.15% potassium chloride and 35 µM butyl-hydroxytoluene followed by centrifugation and recovery of the supernatant. A cocktail of the sample supernatant with 20% acetic acid, 0.8% TBA, milli-Q water and 8.1% sodium dodecyl sulphate was prepared and heated to 95 °C for 30 min, followed by cooling in the dark for 10 min. Once the cooling period was completed, milli-Q water and n-butanol were added to the cocktail, mixed and centrifuged (15 °C, 3000 rpm, 10 min). An aliquot of the supernatant was transferred into a 96-well plate and fluorescence was measured every minute over a 3 min period (λ_ex_ = 515 nm, λ_em_ = 553 nm). The standard curve was prepared using MDA mixed with 1.21 mM 1,1,3,3-tetramethoxypropane.

### 2.6. Statistical Analyses

All the data were subjected to statistical analyses using the Python programming language 3.11.11 (Python Software Foundation https://www.python.org/). Normality and homoscedasticity were initially tested in all the data using the Shapiro–Wilk and Levene’s tests, respectively; when necessary, boxcox transformations were carried out to fulfil the requirements to perform parametric statistics. When normality was not achieved, non-parametric tests were performed. One-way ANOVA tests were performed to determine whether any significant differences amongst the experimental groups existed. When the one-way ANOVA returned significant differences, a *post hoc* Tukey HSD test was performed. For non-normal data, Kruskal–Wallis tests were performed, followed by the Dunn *post hoc* test to determine significant differences amongst groups. A significance of α = 0.05 was employed in all the statistical analyses. Given that we aimed at screening five antioxidants and five phytoplankton species to target the best candidate to support corals against bleaching, conventional ANOVA and non-parametric equivalent tests allowed us to perform multiple comparisons of the biomarkers to determine which antioxidant and/or phytoplankton species offered the best protection to corals.

A heatmap was generated to visualise any patterns of physiological and biochemical responses (i.e., biomarkers) across experimental conditions, allowing the clustering of the conditions using the negative control (Ctrl 26 °C) as the baseline at which corals were at their best physiological state. For this reason, all variables were standardised using z-score normalisation [= (x − µ)/σ)], where x is the obtained value, µ is the mean, and σ is the standard deviation for each variable across all conditions. This normalisation ensured that all the variables measured on different scales contributed equally to the analysis to generate the heatmap. Clustering was only applied to the experimental conditions (rows). Similarity between conditions was quantified using Pearson correlation coefficients, which were converted to a distance metric as 1 − r. The resulting dendrogram was used to organise the conditions according to similarities in their overall response profiles. The measured variables (biomarkers) were not clustered but were displayed in a predefined order based on their biological relevance.

## 3. Results

**Figure 1:**
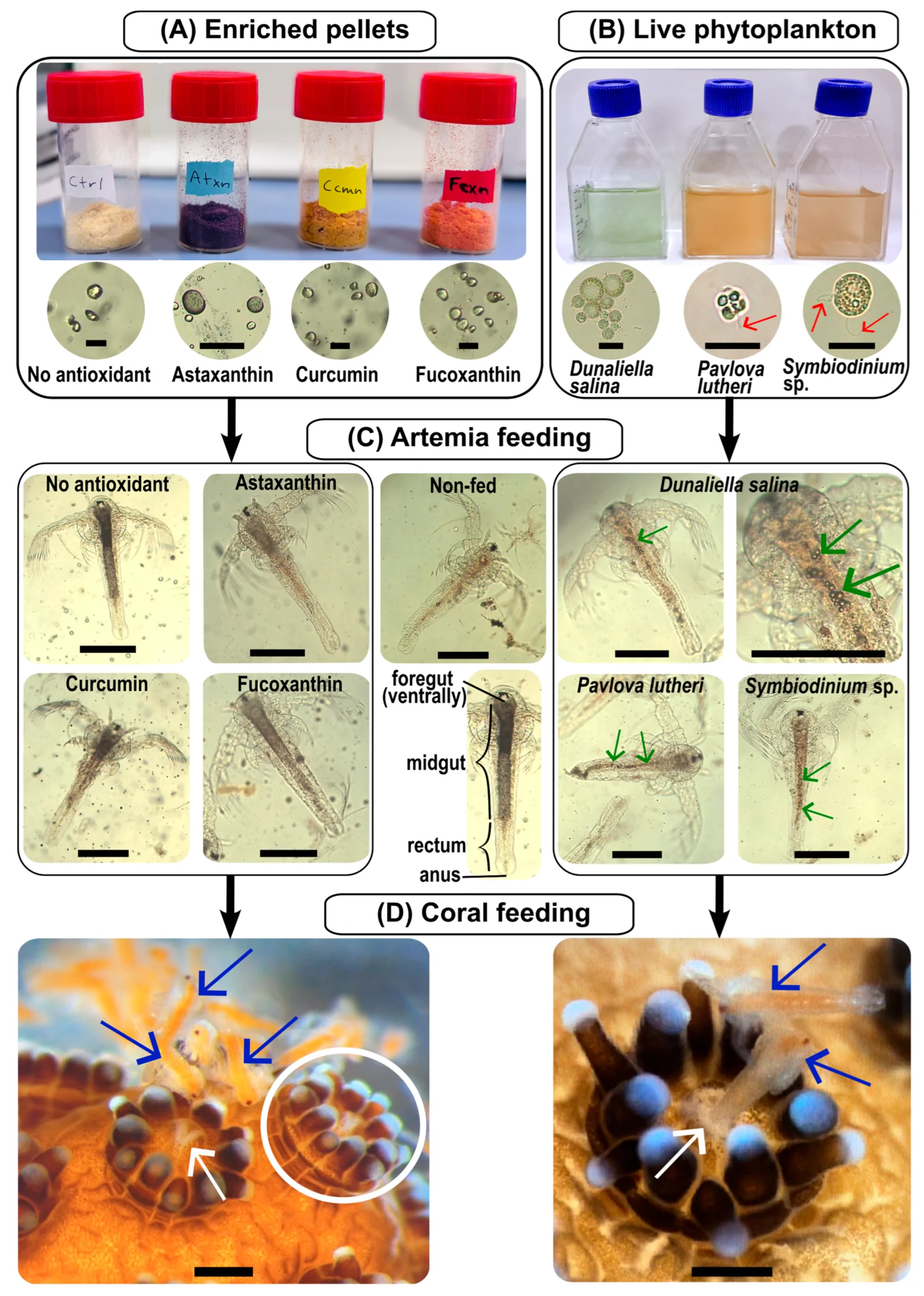
Examples of antioxidant-rich pellets prepared in-house ((**A**); scale bars = 50 µm) and live phytoplankton ((**B**); the red arrows show the algal flagella; scale bars = 10 µm) that were fed to *Artemia salina* ((**C**); the green arrows show the algal cells in the stomach and intestine of *Artemia*; scale bars = 400 µm), and which were fed to the coral *Stylophora pistillata* under a thermal stress experiment ((**D**); the white circle shows a coral polyp with its tentacles, the white arrows the mouth of the polyps and the blue arrows the enriched *Artemia*; scale bars = 400 µm).

The pellets prepared in-house acquired a colouration characteristic of the antioxidant added, except for the control pellets that did not contain any antioxidant. For instance, astaxanthin delivered a purple colour to the pellets, curcumin a yellow colour, and fucoxanthin an orange colour (Figure 1A). By contrast, the pellets containing vitamin C or E had a white and light-yellow colour, respectively, which were similar to their respective purified vitamin formulation. Similarly, the phytoplankton cultures showed a colour typical of the dominant pigments that each of the species possesses (Figure 1B). Upon feeding, the *Artemia* stomach and upper digestive tract (foregut to midgut) looked darker than the lower digestive tract (hindgut or rectum), especially in the *Artemia* that were fed with pellets. Another clear difference between pellet- and phytoplankton-fed *Artemia* was that the latter showed algal cells along the digestive tract when observed under the microscope (Figure 1C). By contrast, *Artemia* that were not fed showed a clearer digestive tract. Our preliminary observations using microscopy confirmed that the corals fed on the enriched *Artemia* (Figure 1D).

**Figure 2:**
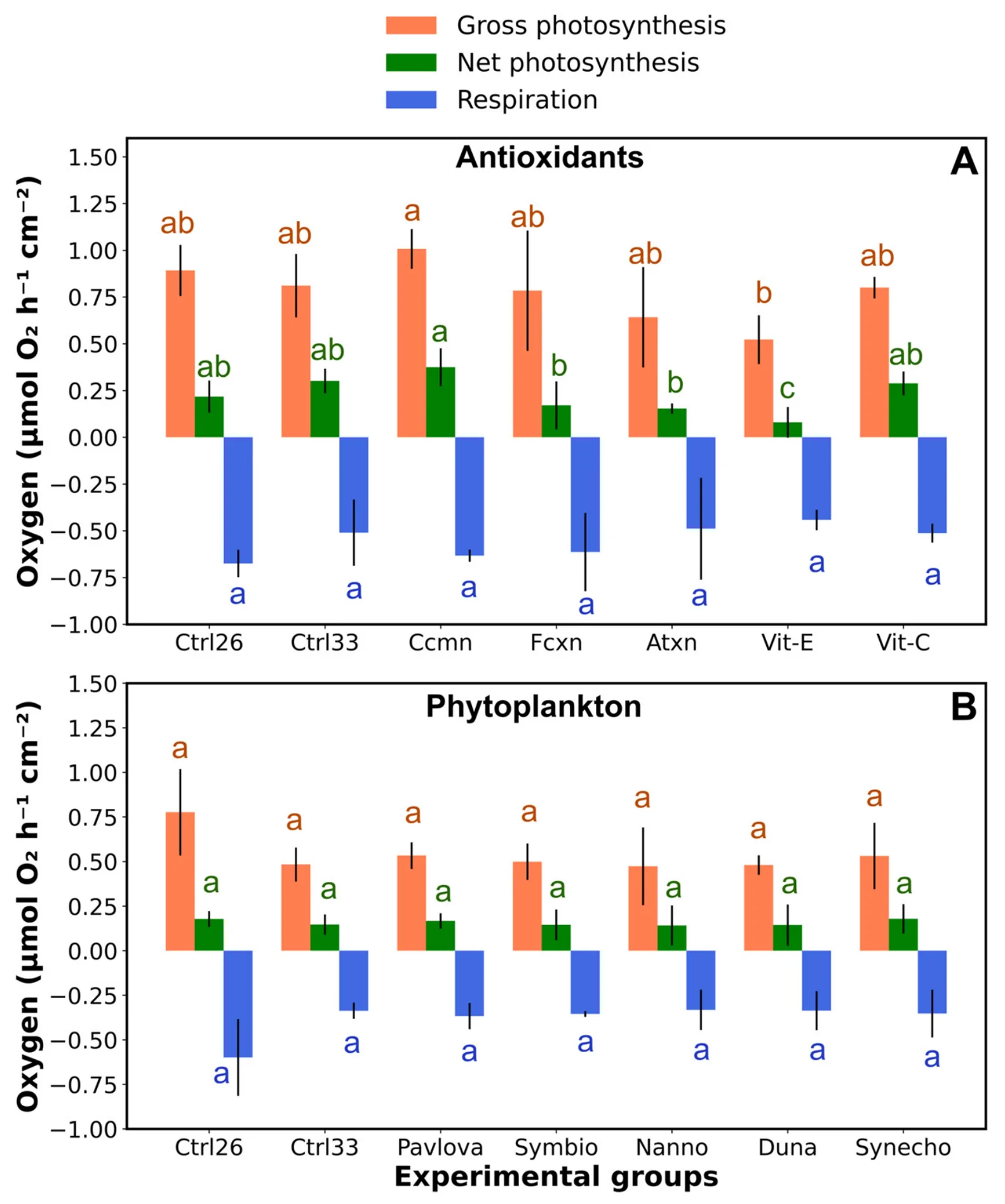
Photosynthesis and respiration of corals under thermal stress while fed with *Artemia* pre-fed with antioxidant-rich pellets (**A**) or live phytoplankton (**B**). Column bars indicate the mean values of replicates (n = 3 for control groups, and n = 5 for groups including phytoplankton and antioxidants), and the black vertical lines indicate the standard deviations. Lower case letters indicate significant differences amongst treatments within the same parameter (Tukey tests, *p* = 0.0101 & *p* ≤ 0.0249 for gross and net photosynthesis, respectively).

Net photosynthesis was lowest in corals fed with *Artemia* enriched with vitamin E compared with all the other antioxidant and control coral groups (0.08 ± 0.08 vs. ≥0.14 µmol O_2_ h^−1^ cm^−2^, respectively). By contrast, the highest net photosynthesis was quantified on corals fed with curcumin-enriched *Artemia* (0.37 ± 0.1 µmol O_2_ h^−1^ cm^−2^). All of the other coral groups were not significantly different from the two controls maintained at 26 and 33 °C (non-thermally and thermally stressed corals, respectively) (Table S1; Figure 2A). On the other hand, corals fed with phytoplankton-enriched *Artemia* did not show any significant differences in gross and net photosynthesis (0.14 to 0.18 µmol O_2_ h^−1^ cm^−2^; ANOVA tests, *p* = 0.2807 & 0.9841, respectively) or respiration (−0.60 to −0.33 µmol O_2_ h^−1^ cm^−2^; Kruskal–Wallis test, *p* = 0.6277) (Figure 2B).

**Figure 3:**
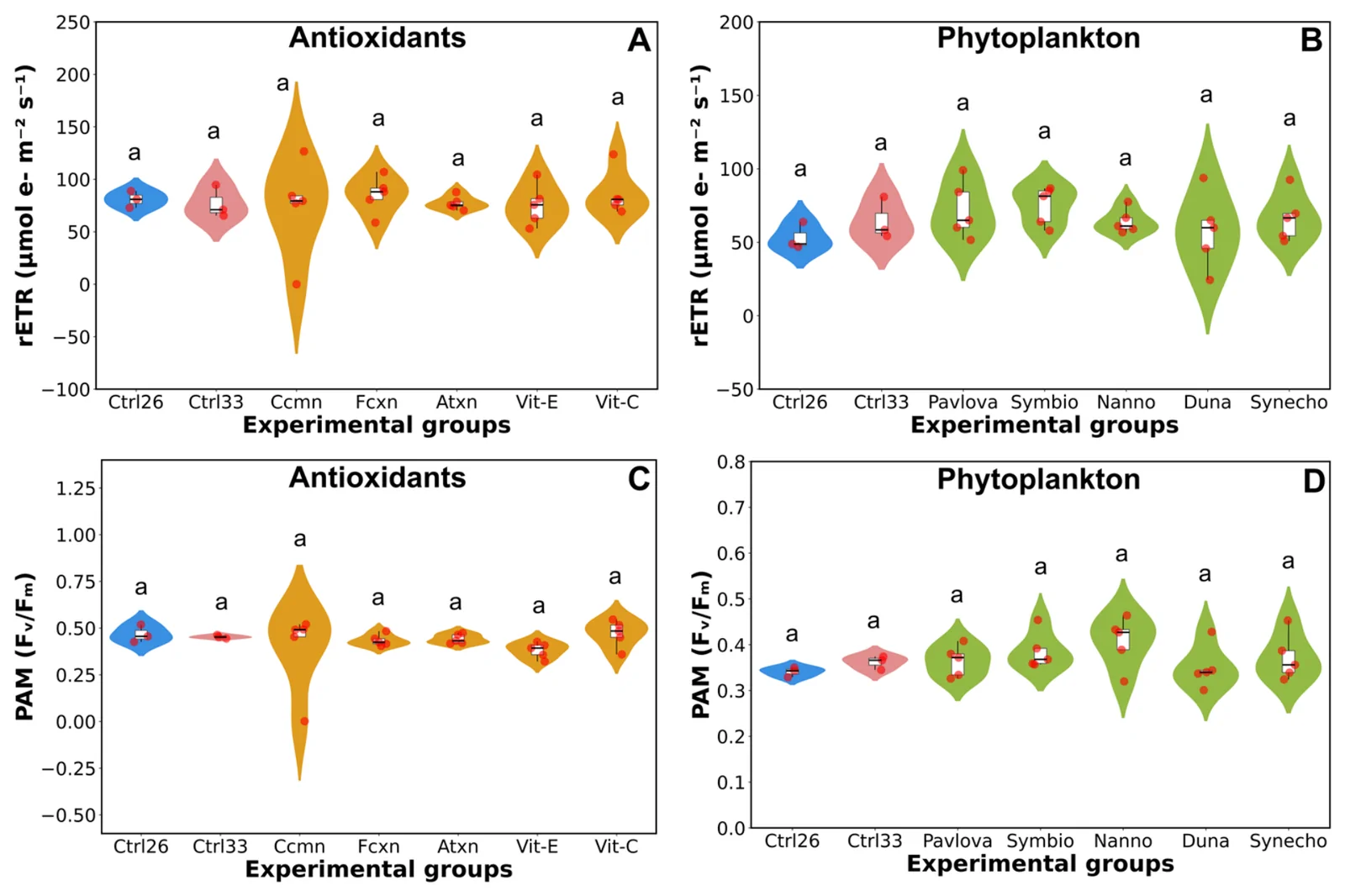
Photosynthetic parameters, including the relative electron transport rate (rETR λ_220_; (**A**,**B**)) and maximum quantum yield efficiency of the photosystem II (*F_v_*/*F_m_*; (**C**,**D**)) in thermally stressed corals and control corals (Ctrl26, control at 26 °C) fed with *Artemia* pre-fed with antioxidant-enriched pellets or phytoplankton. Violin plots with density curves show the distribution of the replicate data points (red dots). The inner box plots show the lower and upper quartiles, with the black horizontal line inside the box showing the median, and the black vertical lines outside the box showing the data distribution.

The relative electron transport rate (rETR λ_220_) and the maximum quantum yield efficiency of the photosystem II (*F_v_*/*F_m_*), did not show any significant differences amongst any of the experimental groups (Kruskal–Wallis tests, *p* = 0.9024 & *p* = 0.1107, respectively, for the antioxidant-rich pellets experiment; ANOVA tests, *p* = 0.5417 & *p* = 0.3786, respectively, for the phytoplankton experiment). Overall, rETR values ranged from 73.47 to 86.09 µmol e^−^ m^−2^ s^−1^ and from 53.17 to 74.99 µmol e^−^ m^−2^ s^−1^ in the antioxidant and phytoplankton feeding coral experiments, respectively (Table S1; Figure 3A,B). Regarding the *F_v_*/*F_m_*, values ranged from 0.39 to 0.47 and 0.34 to 0.41 for the corals fed with antioxidant- and phytoplankton-fed *Artemia*, respectively (Figure 3C,D).

**Figure 4:**
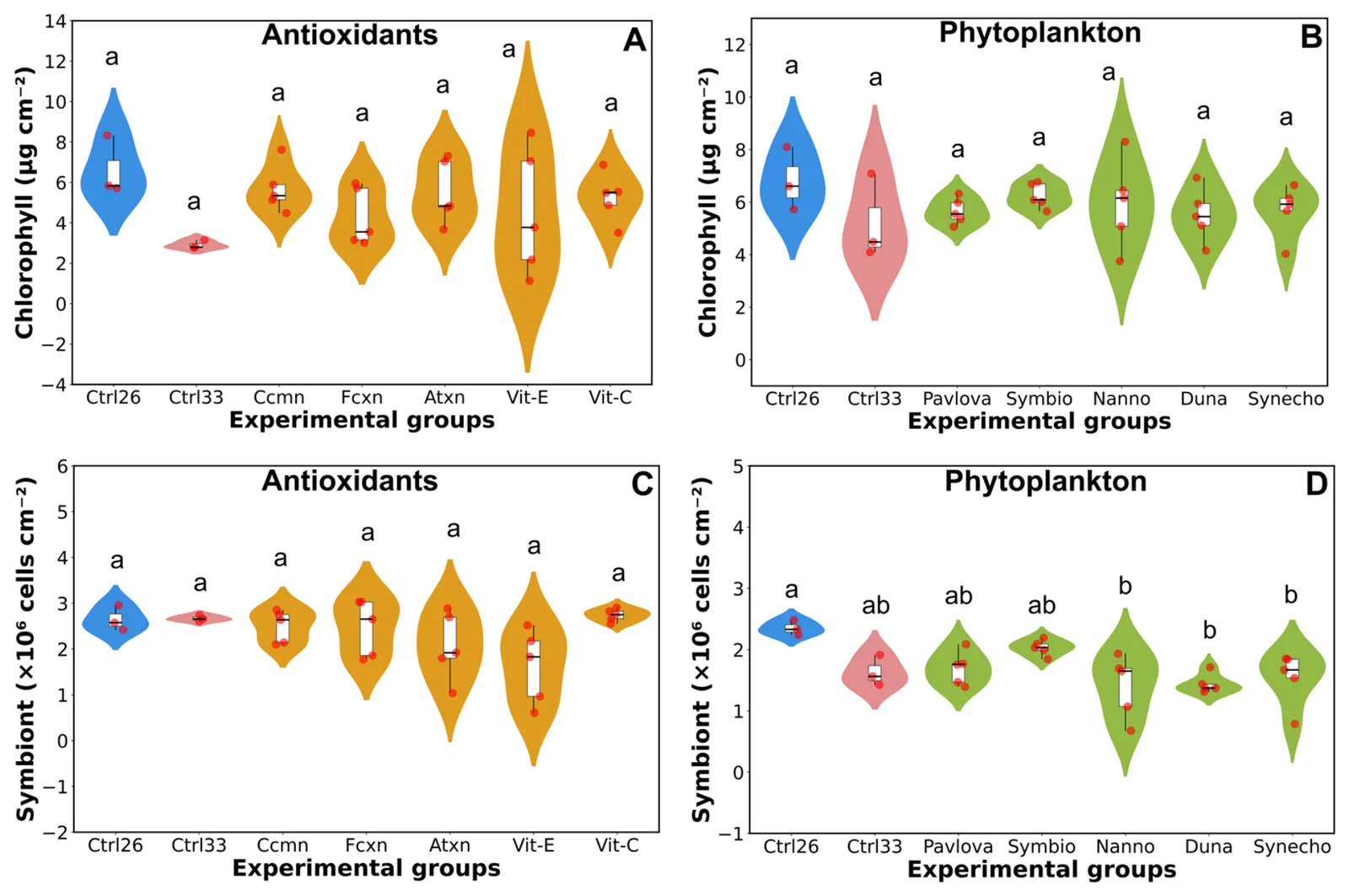
Bleaching parameters of the coral *Stylophora pistillata* upon thermal stress exposure (26–33 °C). Chlorophyll *a* and *c2* (**A**,**B**) and symbiont dinoflagellate (**C**,**D**) densities in non-stressed control (26 °C group, Ctrl26) and thermally stressed control corals (33 °C group, Ctrl33), and corals fed with antioxidant- or phytoplankton-enriched *Artemia*. Violin plots with density curves show the distribution of the replicate data points (red dots). The inner box plots show the lower and upper quartiles, with the black horizontal line inside the box showing the median, and the black vertical lines outside the box showing the data distribution. Lower case letters indicate significant differences amongst the experimental treatments (Tukey test, *p* ≤ 0.0279 in (**D**)).

The two parameters associated with coral bleaching, chlorophyll *a* and *c2* and symbiont dinoflagellate density, did not show significant differences amongst the antioxidants or phytoplankton species tested (ANOVA tests, *p* ≥ 0.0783), except for the symbiont density in corals fed with phytoplankton-enriched *Artemia* (ANOVA test, *p* = 0.0037) (Figure 4). Chlorophyll *a* and *c2* concentrations ranged between 2.91 and 6.63 µg cm^−2^ and 5.22 and 6.81 µg cm^−2^ in corals fed with antioxidant- and phytoplankton-enriched *Artemia*, respectively (Figure 4A,B). The symbiont dinoflagellate density in corals fed with antioxidant-enriched *Artemia* ranged from 1.62 × 10^6^ to 2.74 × 10^6^ cells cm^−2^, with no significant differences observed amongst the experimental groups (Figure 4C). On the other hand, corals fed with *Pavlova lutheri* and *Symbiodinium* sp. maintained their symbiont dinoflagellates, albeit non-significantly from the two controls at 26 °C and 33 °C (Tukey test, *p* ≥ 0.1169). By contrast, *Nannochloropsis* sp., *Dunaliella salina* and *Synechococcus* sp. fed *Artemia* offered significantly less support to corals against symbiont loss when compared with corals maintained at 26 °C (Ctrl26; Tukey test, *p* ≤ 0.0279) (Figure 4D).

**Figure 5:**
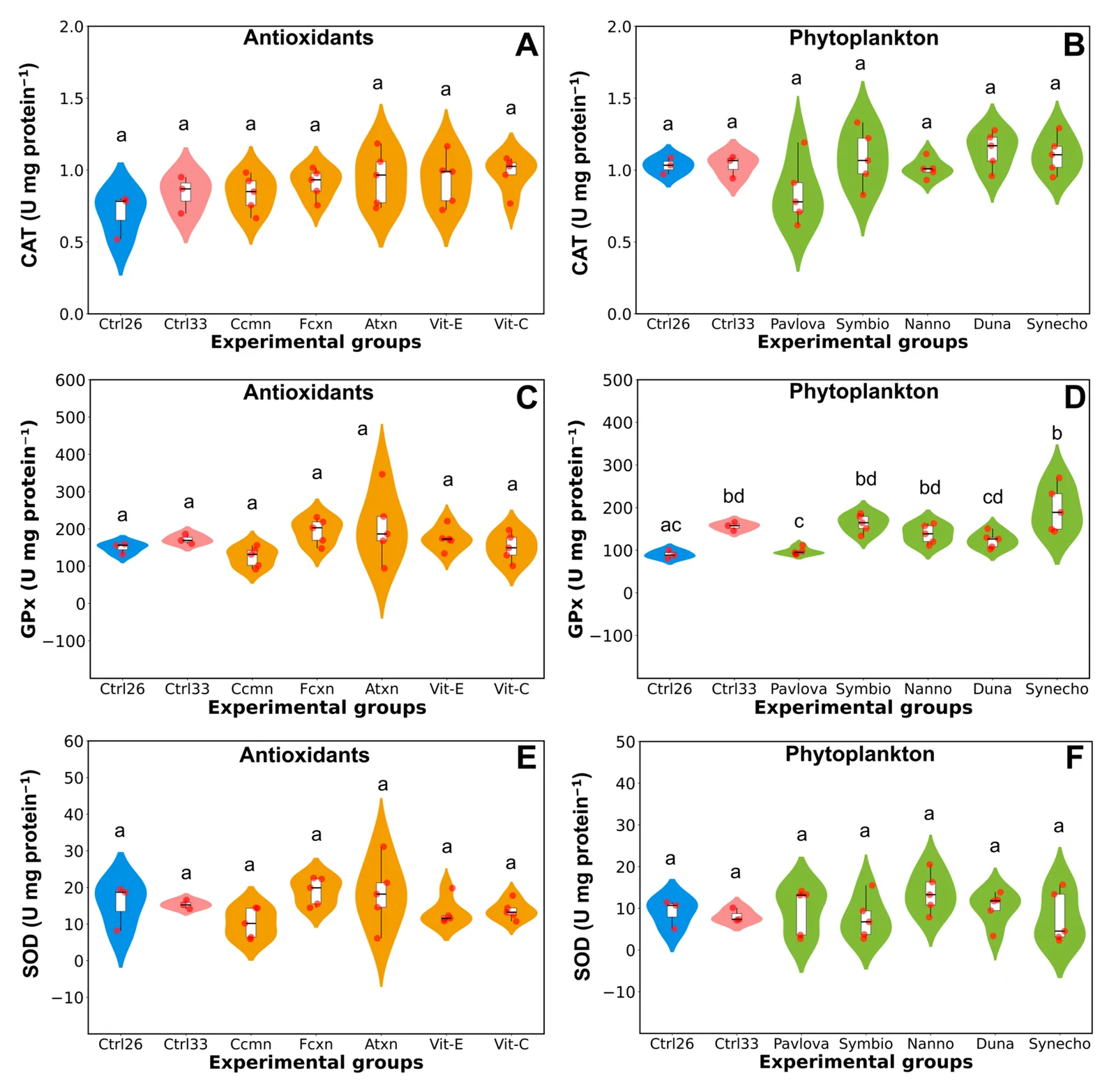
Activity of the antioxidant enzymes catalase (CAT; (**A**,**B**)), glutathione peroxidase (GPx; (**C**,**D**)) and superoxide dismutase (SOD; (**E**,**F**)) in non-stressed (group Ctrl26) and thermally stressed corals (26–33 °C). Violin plots with density curves show the distribution of the replicate data points (red dots). The inner box plots show the lower and upper quartiles, with the black horizontal line inside the box showing the median, and the black vertical lines outside the box showing the data distribution. Lower case letters indicate significant differences amongst the experimental treatments (Tukey test, *p* ≤ 0.0179 in (**D**)).

Antioxidant enzyme activities of catalase and superoxide dismutase (SOD) showed no significant changes in corals across treatments. In contrast, glutathione peroxidase (GPx) activity was significantly altered in some cases, particularly in corals fed phytoplankton-enriched *Artemia*. CAT activity in corals ranged between 0.70 to 0.98 and 0.84 to 1.14 U mg protein^−1^ when given antioxidants and phytoplankton via *Artemia* feeding, respectively (Figure 5A,B; no significant differences observed; ANOVA tests, *p* = 0.2287 & *p* = 0.0875, respectively). GPx activity did not change significantly when corals were fed with antioxidant-enriched *Artemia*, which ranged from 124.79 to 205.79 U mg protein^−1^ (Figure 5C; ANOVA test, *p* = 0.1477). By contrast, corals fed with *Artemia* that were pre-fed with *Pavlova lutheri* showed the lowest GPx activity (97.32 U mg protein^−1^), compared with those fed with *Nannochloropsis* sp., *Symbiodinium* sp., *Synechococcus* sp., and the thermally stressed coral group (Ctrl33) (Tukey test, *p* ≤ 0.0179). Such GPx activity was comparable with the non-thermally stressed corals (Ctrl26; 89.95 U mg protein^−1^; Tukey test, *p* = 0.9809) (Figure 5D). SOD activity ranged between 10.24 to 18.95 and 7.62 to 13.76 U mg protein^−1^ in corals fed with antioxidant- and phytoplankton-enriched *Artemia*, respectively, without significant differences observed amongst the experimental groups (Figure 5E,F; ANOVA tests, *p* = 0.1592 & 0.5159, respectively).

**Figure 6:**
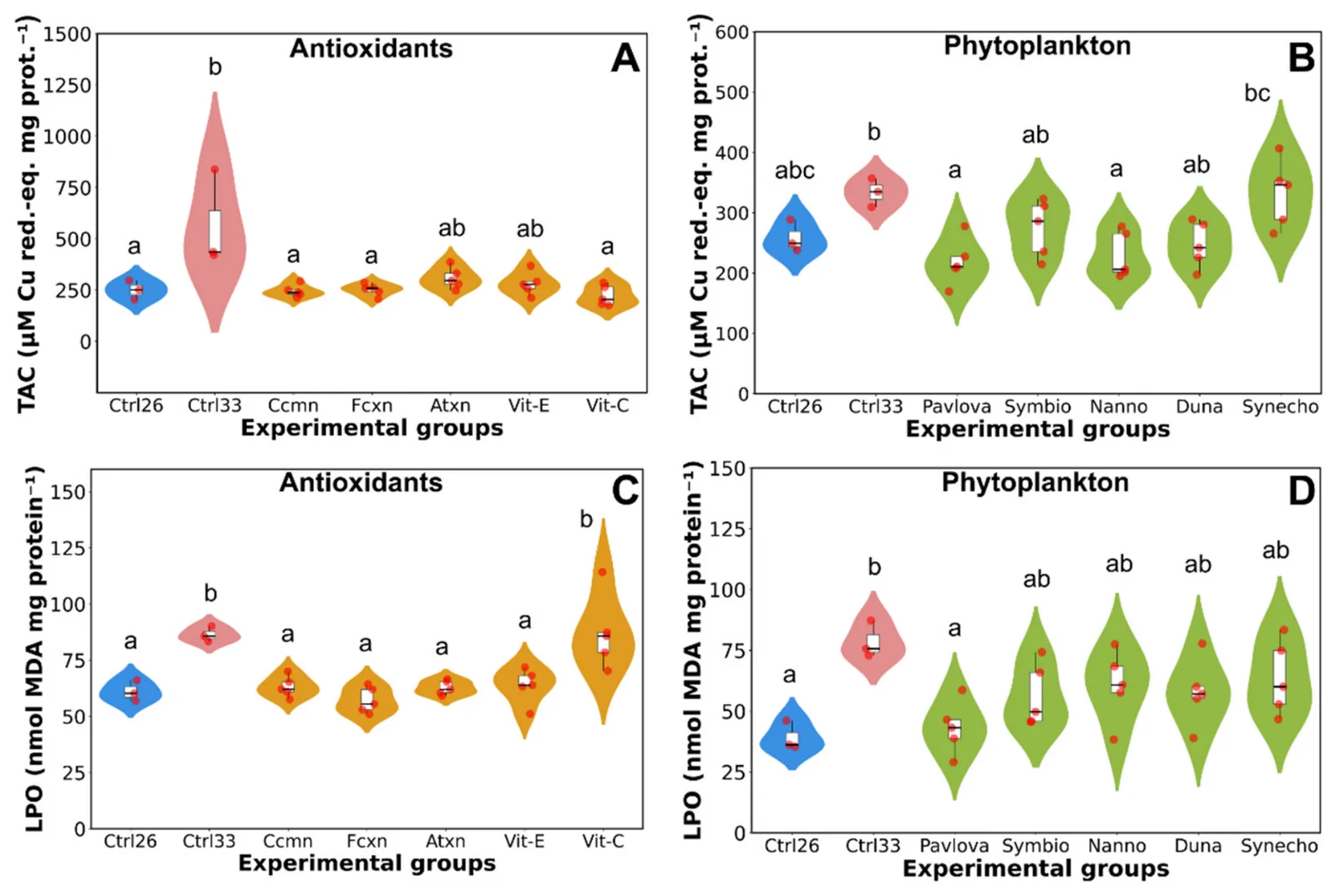
Total antioxidant capacity (TAC; (**A**,**B**)) and lipid peroxidation (LPO; (**C**,**D**)) in non-thermally stressed control (Ctrl26, group at 26 °C) and thermally stressed corals fed with antioxidant- and phytoplankton-enriched *Artemia*. Violin plots with density curves show the distribution of the replicate data points (red dots). The inner box plots show the lower and upper quartiles, with the black horizontal line inside the box showing the median, and the black vertical lines outside the box showing the data distribution. Lower case letters indicate significant differences amongst the experimental treatments (Tukey tests, *p* ≤ 0.0308, *p* ≤ 0.0316, *p* ≤ 0.0329 & *p* ≤ 0.0136 for (**A**–**D**), respectively).

The total antioxidant capacity of corals was highest in the thermally stressed control group (Ctrl33; 565.69 µM Cu reducing-equivalent mg protein^−1^), although it was comparable with the coral groups fed with astaxanthin and vitamin E (308.86 & 281.59 µM Cu reducing-equivalent mg protein^−1^, respectively; Tukey test, *p* ≥ 0.0747). TAC values of corals fed with curcumin, fucoxanthin and vitamin C (223.03 to 251.20 µM Cu reducing-equivalent mg protein^−1^) were significantly lower than the Ctrl33 group (Tukey test, *p* ≤ 0.0161) and comparable with the non-thermally stressed corals (Ctrl26; 251.08 µM Cu reducing-equivalent mg protein^−1^; Tukey test, *p* ≥ 0.7358) (Figure 6A). Regarding the phytoplankton-feeding experiment, corals fed with *Pavlova lutheri* and *Nannochloropsis* sp. showed the lowest TAC (218.62 & 229.16 µM Cu reducing-equivalent mg protein^−1^, respectively), especially when compared with the thermally stressed control corals (Ctrl33; 333.62 µM Cu reducing-equivalent mg protein^−1^) (Figure 6B; Tukey test, *p* ≤ 0.0316). Finally, lipid peroxidation was lowest in non-thermally stressed corals and in those fed with curcumin, fucoxanthin, astaxanthin and vitamin E-enriched *Artemia* (57.15 to 63.64 nmol MDA mg protein^−1^; Figure 6C), as well as in corals fed with *Pavlova lutheri*-enriched *Artemia* (43.27 nmol MDA mg protein^−1^; Figure 6D), especially when compared with the Ctrl33 coral group (Tukey test, *p* = 0.0131).

**Figure 7:**
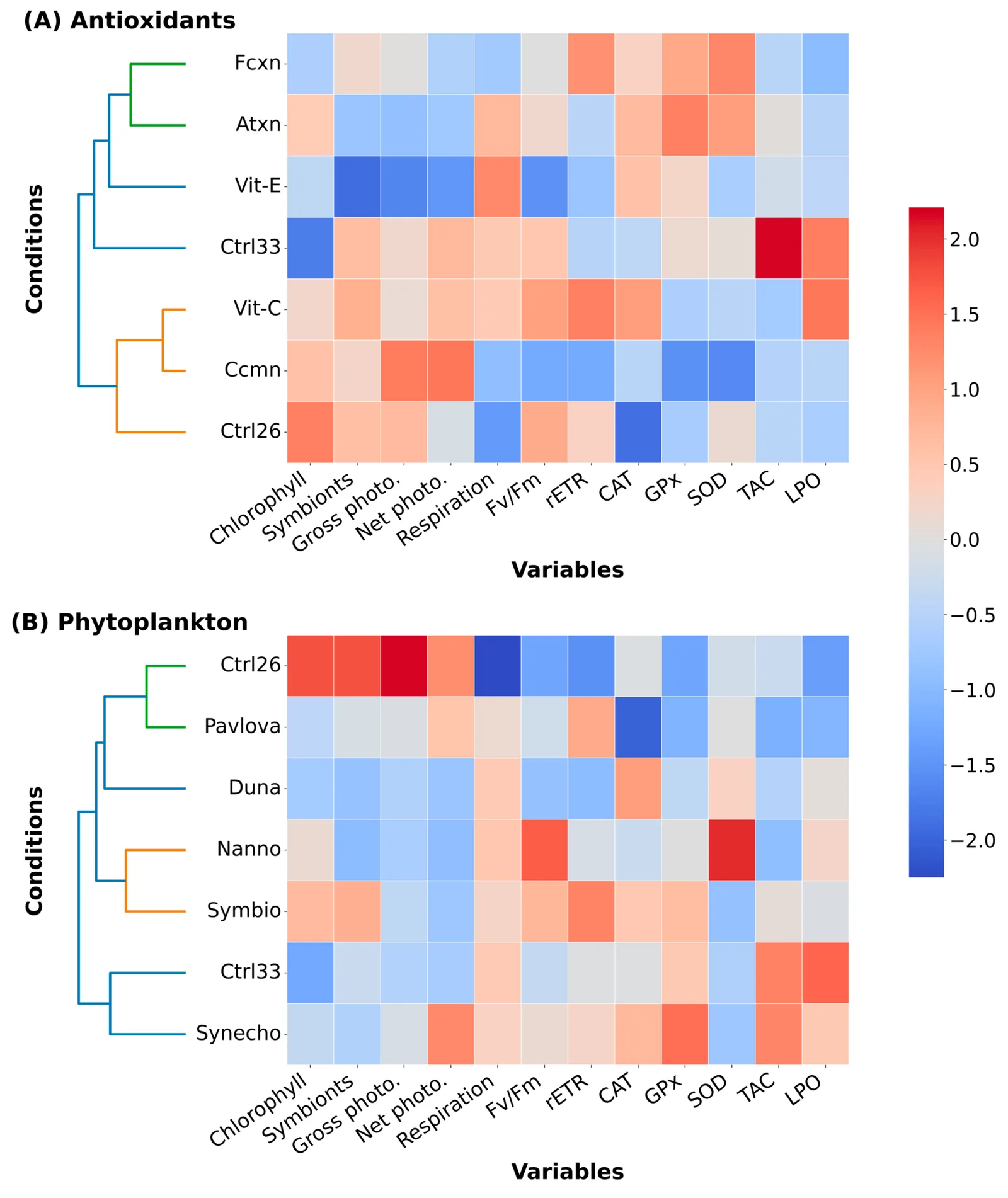
Heatmap of the physiological and oxidative stress biomarker response of *S. pistillata* under thermal stress and unstressed (Ctrl26) while provided with nutritional diets with antioxidants (**A**) and phytoplankton (**B**) via *Artemia* feeding. The experimental groups or conditions were clustered using hierarchical clustering with average association based on correlation distance (1-Pearson correlation) applied after z-score normalisation of the variables. The colours of the cluster lines represent the similarity amongst conditions (i.e., same colour indicates same cluster with similar level of overall biomarker response).

A heatmap and clustered analysis based on similarities in coral responses to thermal stress is presented in Figure 7. Only corals fed with curcumin- and vitamin C-enriched *Artemia* clustered closely with the non-thermally stressed control group (Ctrl26, maintained at 26 °C). However, vitamin C-treated corals exhibited elevated lipid peroxidation (LPO) levels (Figure 6C and Figure 7A), indicating limited reliability of this antioxidant in mitigating oxidative stress. In contrast, corals fed with *Pavlova lutheri*-enriched *Artemia* also clustered with the Ctrl26 group and showed the lowest LPO levels (Figure 6D and Figure 7B). Overall, curcumin and *P. lutheri* emerged as the most effective treatments for reducing oxidative stress and enhancing coral resilience under thermal stress.

## 4. Discussion

The increasing number and duration of marine heatwaves have led to the loss of large areas of tropical coral reefs worldwide [52,53] and have driven some species to functional extinction. This is reflected in the recent case of *Acropora palmata* and *A. cervicornis* in Florida’s coral reef, where >97% mortality was observed during the 2023 marine heatwave [54]. The global coral crisis has intensified the pressure to develop measures to conserve and safeguard coral reefs, including artificial reefs and coral planting and propagation that have been deployed as part of restoration efforts but that remain susceptible to events associated with climate change [20]. Given that 64% of coral reefs are found in developing countries, where economies depend on the reefs for tourism and fishing, and where support for research, development and management is often limited [55], coral restoration and conservation efforts need to be carried out collaboratively between developed and developing nations [56,57]. Therefore, our current study aimed to develop scalable applications for coral conservation interventions, which will be trialled in situ in collaboration with our research partners in Malaysia and Indonesia (https://cordap.org/dipl-team-member/boosting-coral-resilience-with-nutritional-supplements/; accessed on 3 May 2026).

Field-based deployment of biologically active substances is an emerging approach in coral reef intervention strategies. One example includes the application of probiotics (i.e., beneficial microorganisms isolated from healthy corals) over a 5000 m^2^ coral reef area [24]. This intervention has been shown to restructure the coral microbiome and induce associated shifts in the microbial communities of coral-associated fishes and sponges. This field application represents a pilot in situ study exploring the potential for scalable reef interventions, with outcomes benefiting not only corals but broader reef ecosystem health. However, its wider application remains constrained by the need for continuous culturing and reliable availability of probiotic formulations for repeated deployment [58,59,60]. The use of probiotics is based on the premise that beneficial bacteria produce micronutrients, such as iron and vitamins (e.g., biotin, cobalamin, thiamine, riboflavin), which can enhance coral nutrition, growth and resistance against oxidative stress [25,59,61]. These positive effects are particularly important to avoid coral bleaching, given its association with energy deficits from starvation due to (i) the expulsion of the symbiont dinoflagellates that provide corals with most of their energy requirements, and (ii) the scarcity of zooplankton feed during marine heatwaves [15,16]. Therefore, enhancing coral nutrition by providing them with micronutrient supplementation prior to and/or during heatwaves represents a promising strategy for coral conservation.

We recently demonstrated a protective effect of manganese by scavenging superoxide radicals in the ambient seawater of corals, and by leading to the production of manganese oxides in the symbiont dinoflagellates *ex hospite* [31]. Mn oxides are claimed as strong antioxidants that may mimic or activate the endogenous antioxidant defence of a cell, animal or plant, and therefore may confer protection against oxidative stress in corals [31,32,62,63,64]. In this study, we propose an alternative approach for delivering micronutrients to corals during heat stress conditions by using enriched feed. Specifically, we provided corals with zooplankton that had been previously fed with antioxidant-rich diets, including in-house- and low-cost-produced pellets or live phytoplankton. A direct feeding-delivery with the antioxidant-rich pellets was not possible, as microscopic observations showed that *Stylophora pistillata* polyps were not feeding on them. Additionally, the chemical instability of the antioxidants in the pellet matrix, once in contact with seawater, could have compromised their bioactivity due to fast degradation. By contrast, *Artemia*, which are commonly used as feed for mixotrophic corals, were actively feeding on the enriched pellets and phytoplankton cells. These early observations enabled the investigation of a food web approach to coral conservation interventions. This strategy offers a dual nutritional benefit to corals by delivering zooplankton-derived carbon and lipids at higher concentrations than bacteria or phytoplankton alone [65,66,67], while also facilitating the transfer of antioxidants accumulated by *Artemia* from enriched diets. This practice is routinely used in aquaculture, especially during early larval stages of finfish and crustaceans, due to the high nutritional value of zooplankton, such as copepods, rotifers and *Artemia* that are enriched through feeding with phytoplankton and/or already available enriched commercial diets [39,43].

Within this study, the order in which the phytoplankton species offered protection to the coral *S. pistillata* was *Pavlova lutheri* > *Nannochloropsis* sp.~*Symbiodinium* sp. > *Dunaliella salina* > *Synechococcus* sp. On the other hand, the order in which antioxidants supported corals against coral bleaching and oxidative stress was curcumin > fucoxanthin~astaxanthin > vitamin E > vitamin C. The first attempt to use curcumin for coral conservation was reported by Contardi et al. [30], who observed that curcumin contained in biopolymer composites, placed next to *S. pistillata* colonies, offered protection by preventing bleaching and oxidative impacts. They reported that the activity of the antioxidant enzyme catalase was inhibited and significantly lower compared with thermally stressed corals, which did not receive external protection with curcumin. However, the thermal stress exposure with the curcumin composites only lasted 36 h at 29 or 33 °C [30]. However, marine heatwaves typically persist for 5–15 days in tropical regions, 30 days in the northeast and southeast Pacific Ocean, or up to 60 days in the east tropical Pacific, which is highly influenced by El Niño events [68]. Moreover, the Red Sea, where our *S. pistillata* original colonies originated from, is considered one of the warmest seas with marine heatwaves lasting between 7 and 31 days, with temperatures gradually increasing and remaining between 30 and 34 °C, and which have been associated with coral bleaching events [45,46,47,48]. Therefore, it is recommended that coral thermal stress experiments be conducted over several days, with durations of ≥5 days considered representative of a heatwave scenario, as recommended by Hobday et al. [69]. These considerations are critical because the duration and intensity of the heat stress can influence the performance and efficacy of the antioxidant or compound tested for coral protection. For this reason, our heat stress experiment using *S. pistillata* from the Red Sea followed an exposure period of 18 days, during which corals remained at ≥30 °C for 15 days. This experimental length helped demonstrate the efficiency and applicability of curcumin-enriched *Artemia* to protect corals against oxidative stress for longer periods, such as those observed during marine heatwave events.

The observed ranking of antioxidant performance may reflect differences in both trophic delivery efficiency and mechanisms of action. Helgoe et al. [70] proposed that coral bleaching is driven not simply by ROS, but by a complex network of interacting cellular processes including photoinhibition, lipid peroxidation, calcium dysregulation, mitochondrial dysfunction, endoplasmic reticulum stress, and apoptosis [70]. Consequently, antioxidants capable of acting across multiple stages of this cascade would be expected to confer greater protection than compounds that function solely as radical scavengers. The superior performance of curcumin may therefore reflect its broad range of biological activities, including scavenging both reactive oxygen and nitrogen species, suppressing lipid peroxidation, and modulating oxidative stress signalling pathways associated with programmed cell death [71]. Fucoxanthin and astaxanthin likely provided intermediate protection through their carotenoid-mediated quenching of singlet oxygen and peroxyl radicals, thereby protecting photosynthetic and cellular membranes from oxidative damage during thermal stress [72]. Vitamin E, although an effective chain-breaking antioxidant that limits membrane lipid peroxidation, acts over a comparatively narrower range of oxidative processes [73], while vitamin C, a hydrophilic antioxidant, likely exhibited the weakest performance because of both its more restricted intracellular localisation and reduced delivery efficiency through the *Artemia* feeding vector. As vitamin C partitions predominantly into aqueous compartments [73], whereas bleaching-associated oxidative damage is initiated largely within lipid-rich photosynthetic and mitochondrial membranes [74], its protective capacity may have been inherently limited. Furthermore, the lipophilic properties of curcumin, fucoxanthin, astaxanthin and vitamin E likely facilitated their incorporation into soybean oil during pellet manufacture, retention within *Artemia*, and subsequent transfer to corals through heterotrophic feeding. These findings suggest that antioxidant efficacy in feeding-based coral interventions depends not only on intrinsic antioxidant capacity, but also on effective trophic delivery and localisation to the cellular sites where bleaching-associated damage occurs.

We did not observe any significant changes in catalase and superoxide dismutase activity in the coral groups fed with any of the experimental diets. On the other hand, glutathione peroxidase (GPx) activity was significantly lower in corals fed with *Pavlova lutheri*-enriched *Artemia*, compared with thermally stressed control corals (fed with non-enriched *Artemia*). This suggests that the antioxidants and other beneficial compounds produced by *P. lutheri* were accumulated by the *Artemia* and transferred to the corals through feeding, and, consequently, provided antioxidative protection (i.e., lower LPO). *P. lutheri* has been described as a high producer of phytosterols, fucoxanthin, β-carotene and omega-3 polyunsaturated fatty acids, such as eicosapentaenoic and docosahexaenoic acids, and the production of which may vary according to the culturing conditions of the microalga [75,76]. These compounds are commonly targeted by the food and cosmetic industries due to their beneficial properties, such as antioxidant, antimicrobial, anticancer and immunomodulatory bioactivities (reviewed by Vieira et al. [77]). On the other hand, curcumin has been extensively used in human diets, particularly in traditional Asian medicine, due to its anti-inflammatory and antioxidative properties (reviewed by Hewlings & Kalman [71]). Despite the wide knowledge and extensive use of microalgae in aquaculture, and curcumin in human diets and aquaculture, their use as feed supplements has rarely been reported for corals. Therefore, we present for the first time the use of curcumin and *Pavlova lutheri* as feed candidates for coral conservation efforts, with the latter potentially requiring greater investment due to the need to culture and maintain the microalgae. For example, curcumin-based pellets would only require rearing *Artemia* as a live feed, avoiding the two-step production process associated with live microalgal enrichment. This protocol would include culturing the microalgae, a waiting period of a couple of weeks to obtain a high algal biomass, and growing the *Artemia* to be used as feed. Additionally, this approach would also require skilled technical staff, substantial infrastructure and material costs (e.g., temperature-controlled room, autoclave, culturing containers, lights, bioreactors or ponds, nutrients, constant seawater supply) [40,78].

The advantage of our approach is that, once fed, *Artemia* were harvested by filtration (100 µm mesh) and stored frozen until use, with the aim of minimising potential degradation of both the antioxidants and the *Artemia* themselves and to also prevent *Artemia* from metabolising the antioxidants, therefore transferring less of these to the corals. This is a common practice in the aquaculture and aquariology industries, where purchasing blocks of frozen *Artemia* is preferred given the ease of storage and overall handling for feeding [79]. In our case, frozen *Artemia* facilitated coral feeding, which could be transferable to upscaled in situ interventions. For instance, the transportation of harvested frozen *Artemia* could be more reliable and streamlined than transporting tanks with live *Artemia*, which would require large volumes of seawater and aeration. Another advantage that our food web approach can offer to corals under heat stress is that *Artemia* may also provide corals with innate probiotics, although it would have to be delivered to corals as live *Artemia* to keep the probiotics alive. However, biosecurity and cross-contamination factors must be considered given that live *Artemia* can be a vector for disease or infection in aquatic organisms [66,79]. Probiotics have shown benefits to *Artemia* themselves, which, upon feeding with *Bacillus subtilis*, *Lactobacillus plantarum* and *L. lactis*, showed an increased activity of antioxidant enzymes and protection against lipid peroxidation and an increase in survival rate following infection with the pathogenic bacteria *Vibrio anguillarum* [80]. Moreover, the transfer of probiotics via *Artemia* has also been proven in larvae of the Pacific white shrimp *Litopenaeus vannamei*, which showed improved growth and survival upon feeding with *Artemia* enriched with *Bacillus subtilis* and *B. licheniformis* [81].

Based on previous reports, and together with our findings, there is the possibility that enriching *Artemia* with both probiotics and antioxidant-rich diets could deliver a higher level of protection to corals against oxidative stress and subsequent bleaching, although this remains to be tested. However, as with microalgae, the culturing of probiotic bacteria may increase both cost and time investment. Ultimately, a cost–benefit analysis of any proposed coral conservation intervention is required to determine its suitability for real-world applications.

Finally, as many innovative developments for coral conservation continue to arise, it is crucial to determine not only their efficacy when upscaling from a laboratory-developed application to an in situ intervention but also their reliability in terms of cost-effectiveness [82,83,84,85]. Consideration would also be required regarding how associated risks would be identified and managed, and how meaningful consultation and feedback from reef users would be obtained [86]. Amongst the factors that would need to be accounted for are (i) the extension of the coral reef area to be supplemented with enriched *Artemia*; (ii) the amount of pellets to be produced and *Artemia* to be fed; (iii) infrastructure required for *Artemia* feeding, such as fibreglass columns or photobioreactors, aeriation pumps; (iv) overall labour cost, including pellet preparation, *Artemia* growing and feeding, and subsequent field deployment; (v) in situ feeding competition by other organisms; and (vi) long-term ecological impacts, including assessment of coral reef recovery and health. Moreover, emerging automated technologies, such as subsurface automatic feeders or slow-release deployed devices like those employed in aquaculture, could be trialled, which could bypass manual intensive labour, especially for deploying the feed at a large spatial scale [87,88]. Additionally, since we started the feeding of corals as soon as the seawater temperature started to increase, this would translate into deploying the enriched *Artemia* when the onset of a marine heatwave occurs, rather than routine periodic feeding, which would create unnecessary costs. It is recommended that before in situ applications are trialled, ex situ mesocosm studies are carried out as an intermediate step in the upscaling process of this approach. This would allow the assessment of the impacts of the enriched *Artemia* in multi-trophic levels, which would also need to be assessed in situ in the long-term.

## 5. Conclusions

Here, we present curcumin and the microalga *Pavlova lutheri* as promising antioxidant-based interventions for mitigating oxidative stress in corals. Our findings suggest that our low-cost curcumin-enriched pellets prepared in-house and *P. lutheri*, known to produce antioxidants and omega-3 fatty acids, were accumulated by *Artemia* through feeding and transferred to corals. Both curcumin and *P. lutheri* supported *Stylophora pistillata* under thermal stress conditions to fight oxidative stress, which showed a minimal impact on lipid peroxidation and coral bleaching, and which were comparable with non-thermally stressed corals.

We designed this experiment to simulate real-world marine heatwave conditions that have driven mass coral mortality and contributed to the global coral reef crisis. Given the urgent need for coral protection and restoration strategies, our approach represents a potentially scalable intervention for in situ conservation. However, further work at larger, more operational scales, and across additional representative coral species, is required to assess the broader applicability and scalability of this food-web-based approach under changing climatic conditions. In this context, curcumin may offer a more streamlined and cost-effective option than *Pavlova lutheri*, as its application would require only the culture of *Artemia*, rather than combined production of both phytoplankton and zooplankton.

## Figures and Tables

**Table 1:** Dissolution of antioxidants with their respective solvent and conditions for preparation of the pellets to be fed to *Artemia salina*, which was subsequently fed to the coral *Stylophora pistillata* under a thermal stress scenario.

Antioxidant	Product Reference	Final Concentration (*w*/*w*)	Solvent ^a^	Conditions
Curcumin	Sigma, C1386	0.5%	milli-Q water	13.13 mL at pH 7 and 50 °C ^b^
			Soybean oil	0.45 g at 50 °C ^c^
Fucoxanthin	Sigma, F6932	0.062%	milli-Q water	13.13 mL at pH 7 and 50 °C ^b^
			Soybean oil	0.45 g at 50 °C ^c^
Astaxanthin	Sigma, SML0982	0.062%	milli-Q water	13.13 mL at pH 7 and 50 °C ^b^
			Soybean oil	0.45 g at 50 °C ^c^
Vitamin E	Sigma, 258024	0.5%	milli-Q water	13.13 mL at pH 7 and 50 °C
			Soybean oil	0.45 g at room temperature
Vitamin C	Sigma, A4544	0.5%	milli-Q water	13.13 mL at pH 3.5 ^d^
			Soybean oil	0.45 g at 50 °C ^b^

^a^ milli-Q water was used for vitamin C and soybean oil was used for the other antioxidants as they were lipophilic. Both solutions were used in all preparations to maintain uniformity in the recipes. ^b^ To prevent a rapid phase separation when combining milli-Q and soybean oil, which were then added to the dry ingredients and mixed. ^c^ To facilitate dissolution of lipophilic antioxidants, the soybean oil was heated to 50 °C using a stirring/heating plate with continuous mixing using a magnet for 10 min. ^d^ To prevent fast degradation of vitamin C.

## Data Availability

The data and Python codes for the statistical analyses of this study are freely available at https://doi.org/10.5281/zenodo.21640124.

## Supplementary Materials

The following supporting information can be downloaded at https://www.mdpi.com/article/10.3390/antiox15080994/s1: Table S1. Summary of biomarkers assessed on the thermally stressed coral *Stylophora pistillata* and control corals maintained at 26ºC (Ctrl26) while fed with enriched *Artemia salina* which were pre-fed with antioxidant-rich pellets or live phytoplankton. Values show the mean ± standard deviation of each variable..
