# Supplementary material for: Trophic Antioxidant Transfer as a Measure for Scalable Coral Conservation Strategies
Source: Antioxidants (Basel). 2026 Aug 11;15(8):994. doi: 10.3390/antiox15080994 (PMC13509945; doi:10.3390/antiox15080994)
Supplement: Supplementary file 1 [file antioxidants-15-00994-s001.zip › antioxidants-4317024-supplementary.pdf]

Table S1. Summary of biomarkers assessed on the thermally stressed coral *Stylophora pistillata* and control corals maintained at 26ºC (Ctrl26) while fed with enriched *Artemia salina* which were pre-fed with antioxidant-rich pellets or live phytoplankton. Values show the mean ± standard deviation of each variable.

**Condition**	**Gross photosynthesis**[a]	**Net photosynthesis**[a]	**Respiration**[a]	**rETR**[b]	**F_v/F_m**[c]	**Chlorophyll**[d] ***a & c2***	**Symbiont (cells cm⁻²)**	**CAT**[e]	**GPx**[e]	**SOD**[e]	**TAC**[f]	**LPO**[g]
Ctrl26	0.89 ± 0.14	0.22 ± 0.09	-0.67 ± 0.07	80.90 ± 7.99	0.47 ± 0.05	6.63 ± 1.47	2.65 ± 0.28 ×10⁶	0.70 ± 0.16	149.26 ± 14.99	15.46 ± 6.32	251.08 ± 46.61	61.06 ± 4.66
Ctrl33	0.81 ± 0.17	0.30 ± 0.07	-0.51 ± 0.18	77.02 ± 15.56	0.45 ± 0.01	2.91 ± 0.21	2.67 ± 0.07 ×10⁶	0.84 ± 0.13	171.84 ± 13.10	15.31 ± 1.19	565.69 ± 236.16	86.41 ± 3.40
Curcumin	1.01 ± 0.11	0.37 ± 0.10	-0.63 ± 0.03	73.47 ± 45.80	0.39 ± 0.22	5.70 ± 1.18	2.50 ± 0.35 ×10⁶	0.84 ± 0.13	124.79 ± 27.00	10.24 ± 4.11	244.78 ± 30.62	63.17 ± 4.69
Fucoxanthin	0.78 ± 0.32	0.17 ± 0.13	-0.61 ± 0.21	85.19 ± 17.61	0.43 ± 0.03	4.28 ± 1.43	2.47 ± 0.61 ×10⁶	0.91 ± 0.10	194.00 ± 34.73	18.95 ± 3.79	251.20 ± 29.39	57.15 ± 5.78
Astaxanthin	0.64 ± 0.27	0.15 ± 0.03	-0.49 ± 0.27	77.27 ± 6.57	0.44 ± 0.03	5.52 ± 1.58	2.07 ± 0.74 ×10⁶	0.94 ± 0.19	205.79 ± 93.22	18.25 ± 9.16	308.86 ± 52.84	62.75 ± 3.15
Vitamin E	0.52 ± 0.13	0.08 ± 0.08	-0.44 ± 0.05	75.49 ± 19.61	0.38 ± 0.04	4.52 ± 3.14	1.62 ± 0.81 ×10⁶	0.93 ± 0.18	174.16 ± 30.92	13.16 ± 3.75	281.59 ± 57.05	63.64 ± 7.82
Vitamin C	0.80 ± 0.06	0.29 ± 0.06	-0.51 ± 0.05	86.09 ± 21.59	0.47 ± 0.07	5.26 ± 1.22	2.74 ± 0.14 ×10⁶	0.98 ± 0.12	150.80 ± 37.96	13.73 ± 2.61	223.03 ± 50.31	87.24 ± 16.56
Ctrl26	0.78 ± 0.24	0.18 ± 0.04	-0.60 ± 0.22	53.17 ± 9.24	0.34 ± 0.01	6.81 ± 1.20	2.35 ± 0.12 ×10⁶	1.03 ± 0.06	89.95 ± 9.66	9.08 ± 3.49	258.63 ± 26.48	39.25 ± 6.00
Ctrl33	0.48 ± 0.10	0.15 ± 0.06	-0.34 ± 0.05	64.52 ± 14.33	0.36 ± 0.01	5.22 ± 1.63	1.63 ± 0.25 ×10⁶	1.03 ± 0.08	156.37 ± 9.79	8.20 ± 1.66	333.62 ± 23.71	78.65 ± 7.60
Pavlova lutheri	0.53 ± 0.07	0.17 ± 0.04	-0.37 ± 0.07	71.97 ± 19.30	0.36 ± 0.03	5.65 ± 0.50	1.70 ± 0.28 ×10⁶	0.84 ± 0.22	97.32 ± 8.74	9.38 ± 5.69	218.62 ± 39.25	43.27 ± 10.84
Symbiodinium sp.	0.50 ± 0.10	0.14 ± 0.09	-0.35 ± 0.02	74.99 ± 13.11	0.39 ± 0.04	6.24 ± 0.48	2.03 ± 0.13 ×10⁶	1.08 ± 0.20	163.04 ± 21.40	7.62 ± 5.12	274.07 ± 47.36	56.32 ± 13.02
Nannochloropsis sp.	0.47 ± 0.22	0.14 ± 0.11	-0.33 ± 0.11	64.20 ± 8.32	0.41 ± 0.06	5.95 ± 1.69	1.40 ± 0.52 ×10⁶	1.01 ± 0.07	137.89 ± 22.59	13.76 ± 4.91	229.16 ± 38.83	60.56 ± 14.60
Dunaliella salina	0.48 ± 0.05	0.14 ± 0.12	-0.34 ± 0.11	57.73 ± 25.55	0.35 ± 0.05	5.52 ± 1.02	1.44 ± 0.16 ×10⁶	1.14 ± 0.13	123.41 ± 19.16	10.09 ± 4.07	246.99 ± 38.12	57.85 ± 13.85
Synechococcus sp.	0.53 ± 0.19	0.18 ± 0.08	-0.35 ± 0.13	66.76 ± 16.43	0.37 ± 0.05	5.68 ± 0.99	1.54 ± 0.44 ×10⁶	1.11 ± 0.13	197.00 ± 54.13	7.81 ± 6.24	331.90 ± 55.82	63.60 ± 15.29

[a] Gross photosynthesis, net photosynthesis and respiration units are µmol O_2 h^{-1} cm^{-2}

[b] Relative electron transport rate (rETR λ_{220}) units are µmol e^- m^{-2} s^{-1}

[c] Maximum quantum yield efficiency of photosystem II (dimensionless)

[d] Chlorophyll *a* & *c2* units are µg cm^{-2}

[e] Catalase (CAT), glutathione peroxidase (GPx) and superoxide dismutase (SOD) units are U mg $protein^{-1}$

[f] Total antioxidant capacity (TAC) units are µM Cu reduced-equivalent mg $protein^{-1}$

[g] Lipid peroxidation (LPO) units are nmol MDA mg $protein^{-1}$
